# Cytotoxic and cell cycle arrest properties of two steroidal alkaloids isolated from *Holarrhena floribunda* (G. Don) T. Durand & Schinz leaves

**DOI:** 10.1186/s12906-019-2521-9

**Published:** 2019-05-31

**Authors:** J. A. Badmus, O. E. Ekpo, A. A. Hussein, M. Meyer, D. C. Hiss

**Affiliations:** 1 Department of Medical Biosciences, University of the Western Cape, Private Bag X17, Bellville, Cape Town, 7535 South Africa; 2 Department of Chemistry, University of the Western Cape, Private Bag X17, Bellville, Cape Town, 7535 South Africa; 3 Department of Biotechnology, University of the Western Cape, Private Bag X17, Bellville, Cape Town, 7535 South Africa

**Keywords:** *H. floribunda*, Steroidal alkaloids, Cytotoxicity, Cell cycle

## Abstract

**Background:**

The plant *Holarrhena floribunda* (*H. floribunda*; G. Don) is indigenous to sub-Saharan Africa and is traditionally used to treat several ailments. The present study was carried out to isolate and characterize bioactive compounds with anti-proliferative activity present in *H. floribunda* extracts.

**Methods:**

Compounds were isolated from *H. floribunda* using the bioassay-guided fractionation technique of repeated column chromatography and the step-wise application of the MTT reduction assay to assess antiproliferative bioactivity. The structures of the compounds were identified mainly using NMR. The effects of the isolated compounds on the viability, cell cycle and proliferation of human cancer cell lines (MCF-7, HeLa and HT-29) as well as the non-cancerous human fibroblast cell line (KMST-6) were investigated.

**Results:**

Bioassay-guided fractionation yielded two steroidal alkaloids: holamine (**1)** and funtumine (**2**). The MTT reduction assay shows that both compounds exhibited selective dose-dependent cytotoxicity against the cancer cell lines studied. The isolated compounds induced cell cycle arrest at the G_0_/G_1_ and G_2_/M phases in the cancer cell lines with significant reduction in DNA synthesis. The results obtained show that the cancer cells (MCF-7, HeLa and HT-29) used in this study were more sensitive to the isolated compounds compared to the noncancerous fibroblast cells (KMST-6).

**Conclusion:**

The ability of the isolated compounds to cause cell cycle arrest and reduce DNA synthesis raises hopes for their possible development and use as potent anticancer drugs. However, more mechanistic studies need to be done for complete validation of the efficacy of the two compounds.

## Background

Plants are a reservoir of compounds with chemical diversity that provides leads for the discovery of new drugs against human diseases [1]. Folklore medicine as practiced worldwide demonstrates that plants are an important and reliable source of bioactive antitumour, antioxidant and antimicrobial compounds [2]. Assessing plants used in traditional medicine for various biological capabilities necessitates the isolation and characterization of bioactive components as leads for drug development. The African continent lays claim to about 45,000 plant species, of which about 5000 are used for medicinal purposes [3]. Despite the huge potential presented by this number of species, only 83 out of the 1100 potential drugs sourced from the African continent have been selected as candidates for preclinical drug development and screening platforms [3].

Out of the colossal number of 250,000–500,000 world flora species, only 1–10% of plants have been scientifically evaluated [4]. There is a huge gap between the natural flora endowment and human ability to harness the biodervisity for developing drugs, especially against cancer, with minimal or no adverse reactions. The medicinal constituents derived from plants are not only important as therapeutic agents, but could also serve as templates for the synthesis of active drugs with desired therapeutic profiles [5]. This has opened new vistas for delineating the structural importance of bioactive components of plants. A typical example is camptothecin, which has been developed into different analogues (topotecan, irinotecan, belatecan and 9-aminocamptothecin) [6].

*Holarrhena floribunda* (G. Don) is a plant with an approximate height of 17 m and girth of 1 m. The plant belongs to the family of *Apocynaceae* and is commonly found in secondary regeneration in deciduous forests, savanna woodlands in Senegal, Nigeria and in the Congo basin [7]. *Apocynaceae*, the dogane family of flowering plants includes more than 250 genera and 2000 species of trees, shrubs, woody vines and herbs distributed primarily in tropical and subtropical areas of the world [8]. All the members of the family are known to produce abundant milky latex; simple, opposite and whorled leaves; slightly fragrant, colourful and large flowers with five contorted lobes; and with paired fruits [8]. The most common plant-derived compounds with potential medicinal properties are alkaloids, cardiotonic glycosides, saponin, and iridoids [8]. Some bioactive natural products isolated from *Apocynaceae* include vinblastine, quabain, reserpine and ibogaine. The alkaloids of the family have been historically useful to treat cancer with many other compounds awaiting discovery [8]. Some members of the *Apocynaceae* family reported to have anticancer properties include *Nerium oleander* [9], *Alstonia macrophylla* [10]*, Cerbera manghas* [11] and *Calotropis gigantean* [12]. However, based on an ethnomedical survey conducted by Abreu et al. (1999) in accordance with disposition of local healers in the Contuboel region of Guinea-Bissau, *H. floribunda* was screened along with seventeen other plant extracts for antimicrobial, antitumour and antileishmania activity [13]. The extract of *H. floribunda* was found to have significant antitumor effects against KB (squamous carcinoma), SK-Mel 28 (melanoma), A549 (lung carcinoma) and MDA-MB 231 (human breast carcinoma) cell lines [13]. We have also previously reported on the antiproliferative and apoptotic effects of the methanolic leaf extract of *H. floribunda* against HeLa (cervical carcinoma), MCF-7 (breast carcinoma) and HT-29 (colorectal carcinoma) cell lines [14].

The objective of this research was to isolate, purify and characterize the bioactive components of the methanolic leaf extract (MLE) of *H. floribunda,* using bioactivity-guided fractionation and to investigate the cytotoxic effects of the isolated compounds in cancerous (HeLa, MCF-7 and HT-29) and non-cancerous (KMST-6) human cell lines by evaluating the effects of the compounds on cell cycle and DNA synthesis.

## Methods

### Plant material

*Holarrhena floribunda* (G. Don) leaves were collected in Igbajo, Osun State, Nigeria, during the rainy season. It was identified, authenticated and deposited at the Federal Research Institute of Nigeria (FRIN) herbarium with voucher number FHI 10976.

### Chemicals and reagents

Dimethylsulfoxide (DMSO), tetrazolium salt 3-[4,5-dimethylthiazol- 2-yl]-2,5-diphenyltetrazolium bromide (MTT), penicillin-streptomycin, potassium iodide (PI), trypsin, solvents (chloroform, ethylacetate, methanol), paraformaldehyde and RNase were purchased from Sigma-Aldrich (St. Louis, MO, USA). Dulbecco’s Modified Eagle Medium (DMEM) and phosphate-buffered saline (PBS) were obtained from Gibco (USA) and alumina oxide from Fluka (AG, Buch, Switzerland). The ELISA-BrdU kit was purchased from Roche Diagnostic GmbH (Mannheim, Germany).

### Maintenance of cell culture

The cancer cell lines, HT-29 (human colon adenocarcinoma), Hela (human cervical cancer), MCF-7 (human breast adenocarcinoma) and the non-cancerous human fibroblast cell line (KMST-6) used in this study were generously provided by Prof Denver Hendricks (Department of Clinical and Laboratory Medicine, University of Cape Town, South Africa). All cell culture operations were carried out in a model NU-5510E NuAire DHD autoflow automatic CO_2_ air-jacketed incubator and an AireGard NU-201-430E horizontal laminar airflow tabletop workstation that provides a HEPA filtered clean work area (NuAire). The cell lines were maintained in complete DMEM growth medium supplemented with 10% foetal bovine serum (FBS), and 1% penicillin/streptomycin (100 U/ml penicillin and 100 μg/ml streptomycin). The cells were maintained as monolayer cultures at 37 °C in a humidified incubator (relative humidity 80%) in an atmosphere of 5% CO_2_ and 95% air.

### Fractionation of methanolic leaf extract (MLE) of *Holarrhena floribunda*

Pulverized plant material (1.75 kg) was extracted with methanol (5 L) as previously reported [15]. The crude extract obtained (175 g) was fractionated using a silica gel packed column, eluted with a gradient mixture of increasing polarity using hexane: ethyl acetate (10:0), (9:1), (7:3), (5:5). (3:7), (00:10) followed by ethyl acetate: methanol mixture (9:1), (8:2), (6:4), (4:6). A total of 51 fractions (500 ml each) was collected. Similar fractions after being sprayed with vanillin-sulphuric acid and Dragendorff reagents were combined to yield 18 main fractions.

### Bioassay-guided fractionation

The 18 main fractions obtained from the MLE were subjected to bioassay-guided fractionation using the (MTT) reduction assay to identify fractions that contain cytotoxic compounds which could potentially be isolated by further sub-fractionation.

### Isolation of cytotoxic compounds

The bioassay-guided fractionation of 18 fractions led to the identification of 3 fractions with significant cytotoxic activities. Based on their TLC profiles, 3 fractions (15, 16 and 17) were found to contain the same active compounds. Fraction 17 (0.6 g) was subjected to alumina oxide (Fluka AG, Buch SA) Fluka type 507C neutral packed column chromatography eluted with a gradient mixture of 1–10% dichloromethane/ methanol to yield pure compounds **1** (49.5 mg, 0.00028% dry weight) and **2** (22.2 mg, 0.00013% dry weight). The characterization and structural elucidation of compounds with significant cytotoxic effects were performed using nuclear magnetic resonance (NMR). The NMR spectra (^1^H and ^13^C NMR) were recorded in CDCl_3_ on a 200 MHz Varian Unity Inova spectrometer (Gemini 2000) housed at the Department of Chemistry (University of the Western Cape). The identification was completed after the comparison of the obtained data with previously published work.

### MTT reduction assay

Viable cells were seeded at a density of 5 x10^4^ cells/ml (100 μl/well) in 96-well plates and incubated in a humidified atmosphere of 5% CO_2_ and 95% air at 37°C for 24 h to form a cell monolayer. After 24 h, the cell culture medium was aspirated and 100 μl fresh medium containing varying concentrations of test compounds in log_10_ doses were added to the cells and the 96-well plates incubated for a further 24 h [16, 17].

After the 24 h treatments, 20 μl of 5 mg/ml MTT (prepared in PBS) was added to each well and the 96-well plates incubated for 3 h at 37°C in a 5% CO_2_ atmosphere. The supernatant was aspirated and 150 μl of isopropanol added to each well after which the plate was gently shaken for 15 min to solubilize the formazan crystals and the absorbance was measured at 560 nm using a Glomax Multi Detection System (Promega, USA). The percentage inhibition of cell proliferation was calculated using the formula below and IC_50_ values computed by non-linear regression analysis of log dose-response sigmoidal curves using GraphPad Prism version 6.05 for Windows (GraphPad Software, La Jolla California USA, www.graphpad.com).

$$ \%\mathrm{Inhibition}\ \mathrm{of}\ \mathrm{Proliferation}=100\hbox{-} \frac{\mathrm{Test}\ \mathrm{OD}}{\mathrm{Untreated}\ \mathrm{OD}}\mathrm{x}\ 100\% $$

### Cell cycle analysis

Cells were seeded at a density of 2 × 10^5^ cells/well in 6-well plates and incubated for 24 h at 37 °C in a CO_2_ incubator to form a monolayer. The cells were treated for 12 and 24 h with the isolated compounds at concentrations equivalent to the IC_50_ concentrations (as determined by the MTT reduction assay). After treatment, the cells were detached with trypsin, washed in PBS and the cell pellet was resuspended in 1 ml of 1% paraformaldehyde and placed on ice for 30 min. Ethanol (70%, v/v) was slowly added to the cells while the sample was vortexed to reduce cell clumping. The cells were stored at − 20 °C for 48 h, after which the cells were recovered by centrifugation at 4000 rpm for 10 min. The cell pellets were washed twice in PBS and then resuspended in 1 ml PBS containing 100 μg/ml RNase and 40 μg/ml propidium iodide. Cell cycle phase distribution was determined using a FACS Calibur Flow Cytometer (BD Biosciences Franklin Lakes, NJ, USA). The DNA content of 50,000 events was determined by ModFit software (Verity Software House, Topsham, ME), which provided histograms to evaluate the cell cycle distribution [14].

### Bromodeoxyuridine (BrdU) incorporation assay

Cells were seeded at a density of 5 × 10^4^ cells/well in black-walled 96-well microtiter plates and incubated for 24 h at 37 °C in a CO_2_ incubator to form a monolayer. The cells were treated for 12, 24 and 48 h with the isolated compounds at concentrations equivalent to the IC_50_ concentrations (as determined by the MTT reduction assay). Cell proliferation was determined using the ELISA-BrdU (5-bromo-2′-deoxyuridine) chemiluminescence assay kit (Roche, Germany), according to the manufacturer’s instruction, and expressed as the percentage DNA synthesis relative to the untreated control.

### Statistical analysis

The data were analyzed by Two-Way ANOVA, followed by Tukey’s multiple comparisons test and IC_50_ values estimated from non-linear regression curves were calculated using GraphPad Prism software version 6.05 for Windows (GraphPad Software, La Jolla California USA, www.graphpad.com).

## Results

### Bioassay-guided isolation of cytotoxic compounds

**Fig. 1:**
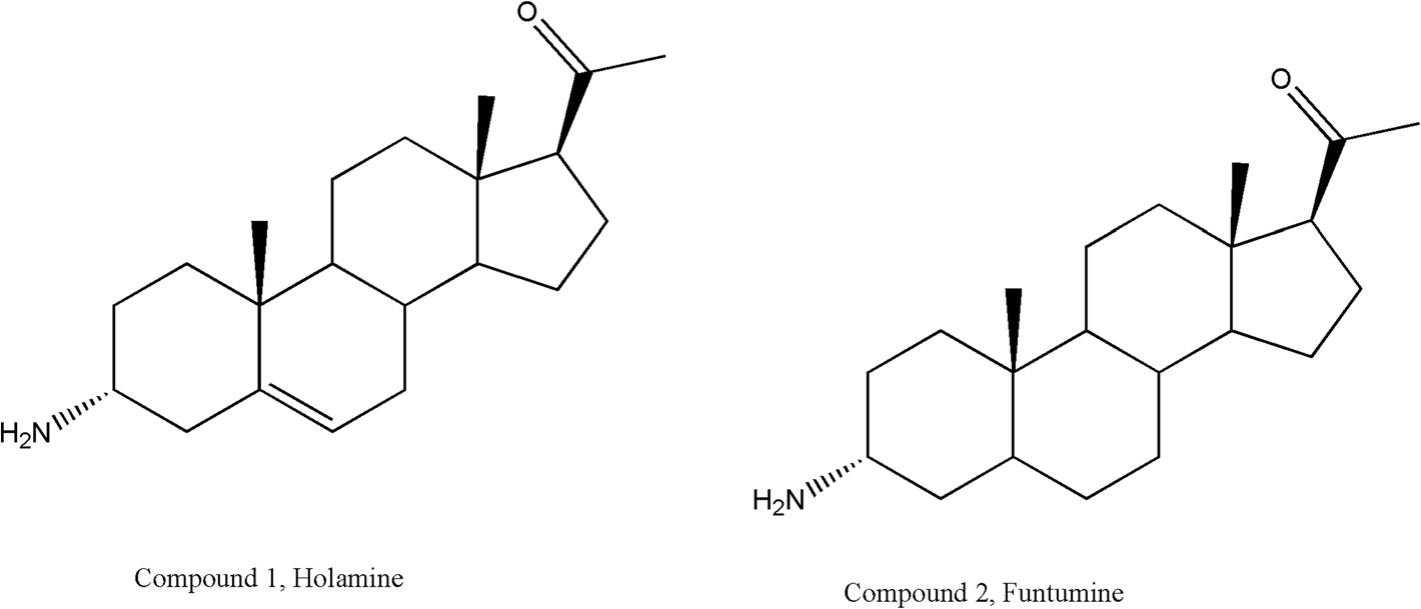
Structures of isolated steroidal alkaloids from the methanolic leaf extract of *Holarrhena floribunda*

Crude extract (175 g) was subjected to silica gel packed column chromatography and yielded 18 fractions, three of which showed the same TLC profile and were cytotoxic. Fraction 17, one of the cytotoxic fractions (0.6 g), subjected to alumina oxide packed column chromatography, yielded 2 pure compounds tagged compounds **1** and **2** with 49.5 mg (0.00028% dry weight) and 22.2 mg (0.00013% dry weight), respectively. The NMR spectral data of the two compounds and their structures are presented in Table 1 and Fig. 1 respectively.

**Table 1:** NMR spectral data for compounds 1 and 2

Chemical shift (ppm)				
	Compound 1		Compound 2	
Peak	^1^H (δ_H_)	^13^C (δ_C_)	^1^H (δ_H_)	^13^C (δ_C_)
1		33.0		32.0
2		29.4		29.8
3	3.12	46.7	3.15	45.2
4		40.0		35.4
5		139.0		39.0
6	5.38	122.8		28.5
7		31.74		28.8
8		31.78		35.9
9		50.2		54.2
10		37.3		36.3
11		20.7		20.7
12		38.7		39.0
13		43.9		44.2
14		56.8		56.7
15		24.4		24.3
16		22.7		22.7
17	2.54	63.6	2.48	63.8
18	0.63	13.1	0.55	13.4
19	0.97	18.8	0.73	11.3
20		209.6		209.8
21	2.10	31.5	2.06	31.9

### Cytotoxicity of isolated compounds

**Fig. 2:**
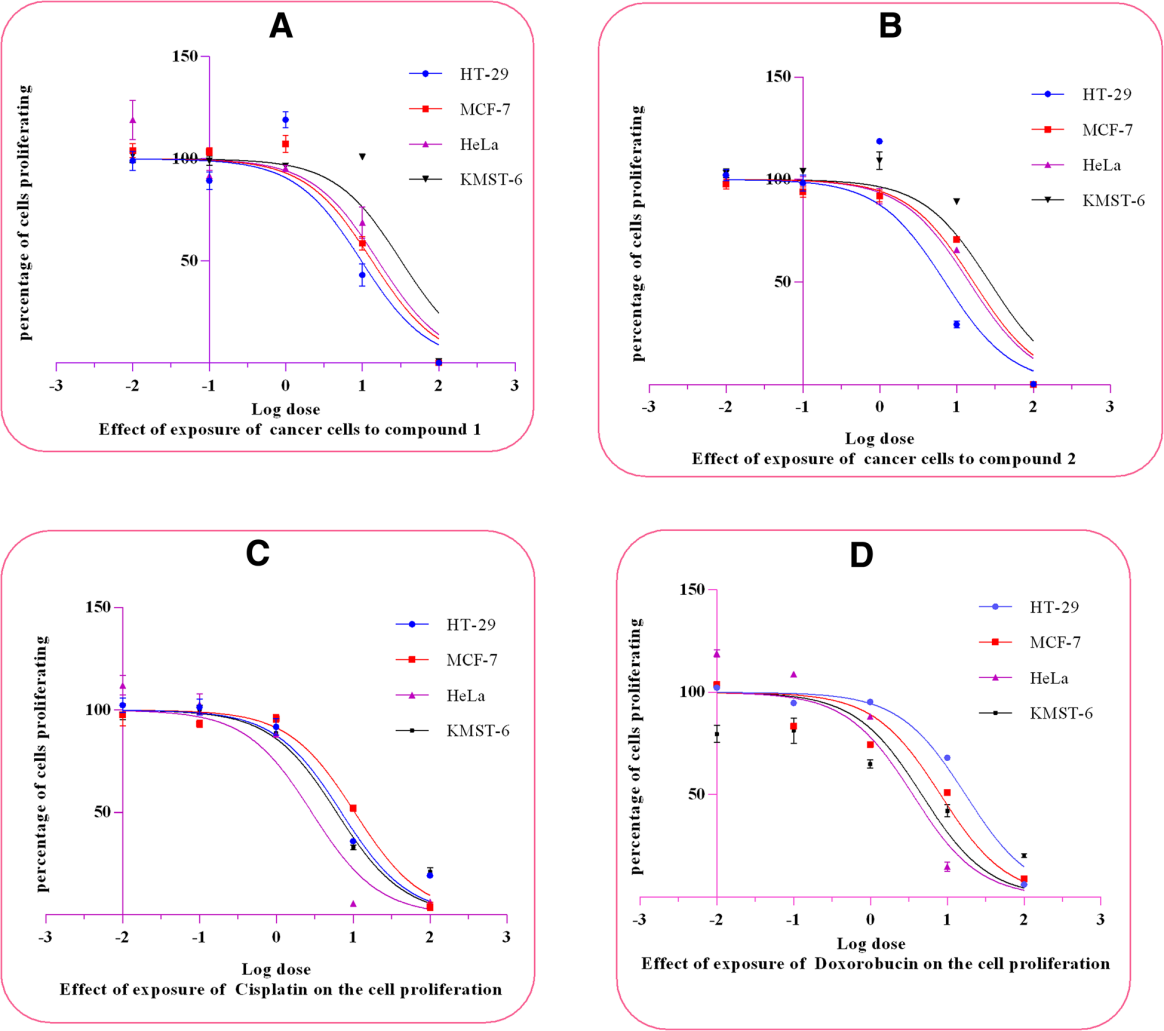
**a**-**d**: Log dose concentrations of isolated compounds and standard drugs on the proliferation of cancer cells

The cytotoxicity of isolated compounds **1** and **2** were tested on HT-29 (colon cancer), MCF-7 (breast cancer), HeLa (cervical cancer) and KMST-6 (non-cancerous fibroblast cells) as presented in Fig. 2. Table 2 shows the IC_50_ values and the selectivity index (SI) of the isolated compounds and standards. The cytotoxicity data showed that compound **1** induced significant cytotoxicity on HT-29 cancer cells compared with the other cancer cell lines with the lowest IC_50_ of 31.06 μM followed by MCF-7 (42.82 μM), HeLa (51.42 μM) and KMST-6 (102.95 μM). The cytotoxic effect of compound 1 is closely similar to that of compound 2. HT-29 was more sensitive to compound **2** with an IC_50_ of 22.36 μM followed by HeLa (46.17 μM), MCF-7 (52.69 μM) and KMST-6 (85.45 μM). The SI for compounds **1** and **2** (3.82 and 3.31, respectively) on HT-29 cells also confirms that this cell line was more sensitive to these two compounds. Cisplatin and doxorubicin showed significant indiscriminate cytotoxicity against cancer cells and the noncancerous cell line while the selectivity index imply that the isolated compounds were more selective towards cancer cells than the noncancerous cell line as opposed to the standard anticancer drugs with a lower selectivity index (Table 2).

**Table 2:** IC_50_ values and selectivity index of compounds (**1** and **2**), cisplatin and doxorubicin treated cancer cells

Cell line	Drug (IC_50_ (μM))				Selectivity index			
	C1	C2	Cisplatin	Dox	C1	C2	Cisplatin	Dox
HT-29	31.06	22.36	7.07	17.38	3.31	3.82	0.87	0.27
HeLa	51.42	46.17	2.91	3.57	2.00	1.62	2.11	1.31
MCF-7	42.82	52.69	10.58	8.20	2.00	1.85	0.58	0.57
KMST-6	102.95	85.45	6.14	4.68				

*C1* Compound 1, *C2* Compound 2, *Dox* Doxorubicin. IC_50_ values were determined by non-linear regression of MTT assay dose-response data

### Effect of isolated compounds on cell cycle progression

**Fig. 3:**
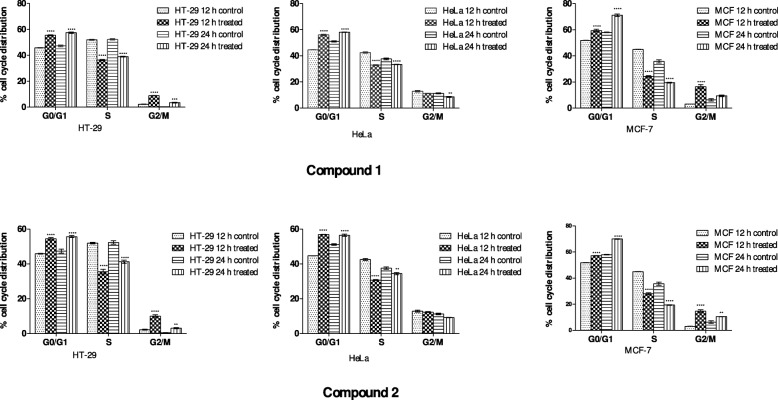
Effects of compounds 1 and 2 on cell cycle progression of HT-29, HeLa and MCF-7 cells stained with PI and evaluated using flow cytometry. The significant levels are indicated with supescripts * * * *P < 0.0001, * * *P < 0.001 and * *P < 0.01

The effects of compounds 1 and 2 on cell cycle progression of cancer cells were evaluated by flow cytometry. The cells were treated with compounds 1 and 2 at concentrations equivalent to the IC_50_ values (Table 2) and a comparative analysis of the percentage cells in the G_0_/G_1_, S and G_2_/M cell cycle phases were performed at 12 and 24 h time points (Figure 3). In general, when the cells were treated with compounds 1 and 2, a significant increase in the percentage of cells in G_0_/G_1_ and G_2_/M phases was observed, which was accompanied by a simultaneous significant decrease in the percentage of cells in the S-phase. The results showed that the compounds induced a significant increase in cell populations in G_0_/G_1_ (*P*<0.01) and G_2_/M (*P*<0.001) phases with concomitant reduction in S-phase of HT-29 cell at both time points. A significant increase was observed in the G_0_/G_1_ phase with reduction in S-phase in HeLa cells treated with both compounds while significant decrease (*P*<0.01) in G_2_/M-phase was only observed at 24 h for compound 1. Both compounds induced significant (*P*<0.0001) increase and decrease in MCF-7 cell populations in G_0_/G_1_ phase and S-phase, respectively. The treatment of MCF-7 cells with compounds 1 and 2 resulted in a significant increase (*P*<0.001) in the G_2_/M-phase at 12 h while at 24 h significant increase induced by compound 2 reduced to (*P*<0.01) while compound 1 G_2_/M-phase increase was not significant.

### Effects of the isolated compounds on DNA synthesis

**Fig. 4:**
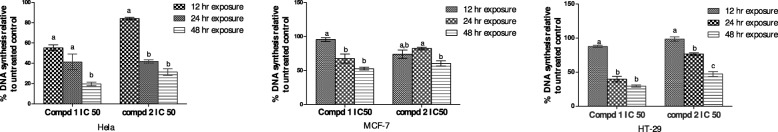
Effects of IC50 concentrations of compounds 1 and 2 on DNA synthesis at 12, 24 and 48 h based on the chemiluminescent BrdU ELISA assay. Bars with non identical letters are significantly different from each other

The effects of the isolated compounds on DNA synthesis were tested using a BrdU chemiluminescent ELISA kit by treating the cells (HeLa, MCF-7 and HT-29) with the respective IC_50_ concentrations determined for the periods of 12, 24 and 48 h in relation to untreated control cells. Figure 4 shows that the two compounds significantly reduced DNA synthesis in all the cell lines. Compound **1** showed a significant reduction (*P* < 0.0088) between 24 and 48 h while compound **2** displayed significant reductions (*P* < 0.0001) in DNA synthesis between 12 and 24 h in HeLa cells. MCF-7 cells showed a significant reduction (*P* < 0.0030) in DNA synthesis when treated with compound 1 between 12 and 24 h while compound 2 significantly (*P* < 0.05) reduced the DNA synthesis between the 24 and 48 h exposure times. Compound **1** only showed a significant reduction (*P* < 0.0001) at 12 and 24 h treatments in HT-29 cells while compound **2** showed a significant reduction between 12 and 24 h (*P* < 0.05) and between 24 and 48 h (*P* < 0.001).

## Discussion

Bioassay-guided isolation of compounds from the cytotoxic fractions of *Holarrhena floribunda* produced in two major aminosteroidal compounds identified as holamine and funtumine. These two steroidal alkaloids were identified based on their NMR spectra (^1^H and ^13^C) which were compared with the existing profiles in the literature [18]. Holamine is a major steroidal alkaloid from the genus *Holarrhena* which was identified in almost all the isolates generated from this genus [18, 19]. To the best of our knowledge, this is the first time the funtumine is isolated from *Holarrhena floribunda* leaves. This compound has been previously isolated from *Holarrhena* and *Funtumia* genera such as *Holarrhene febrifuga, Holarrhena wulfsbergii* and *Funtumia latifolia* [20, 21]. Alkaloids are widely distributed secondary metabolites derived from plants and well-known for their diverse pharmacological efficacies [22]. Fungicidal, anti-inflammatory and antibacterial activities of aminosteroids have been previously described [23, 24]. In addition, steroidal alkaloids are useful as starting materials for the synthesis of pharmaceuticals or important semi-synthetic drugs [25]. Holamine derivatives were used as starting material for the synthesis of immune stimulating aminopregnenes-I and active adjuvant anaphylactic glycinamide-II [26]. A funtumine derivate substituted with a guanylhydrazone moiety has previously been shown to interact selectively with the telomeric G-quadruplex in vitro [27]. Apart from being candidates for anticancer drug discovery, a previous study tends to suggest that steroidal alkaloids could also be used as starting materials and templates for the synthesis of anticancer drugs with desirable characteristics [27].

The cytotoxic activities of the isolated compounds were evaluated using the MTT reduction assay. It is a widely applied assay to evaluate cytostatic and/or cytotoxic potential of chemical and medicinal agents [28]. The cytotoxic effects of these compounds against cancer cell lines (HT-29, MCF-7 and HeLa) compared to non-cancerous fibroblast cells (KMST-6) as presented in Table 2 imply that they are significantly selective towards the cancer cells compared with the non-cancerous cells. The standard anticancer drugs (cisplatin and doxorubicin) are poorly selective between cancerous and non-cancerous cells when judged against the isolated compounds. The assay showed that HT-29 is more sensitive to the cytotoxic effects of both compounds 1 and 2 with IC_50_ values of 31.06 and 22.36 μM, respectively. The IC_50_ values for both compounds in HeLa, MCF-7 and KMST-6 cells were higher than 40 μM. This is the first cytotoxicity reported on the isolated compounds against this panel of cancer cells. The cytotoxic effects of compound 1 isolated from *Holarrhena curtisii* on HL-60 (human promyelocytic leukemia) and P-388 (murine lymphocytic leukemia) have been reported previously [18], while compound **2** displayed no cytotoxicity at a neuro-active concentration [29].

However, many anticancer agents are reported to play roles in cell cycle arrest and cell death [30]. Cell cycle transitions were evaluated in this study using flow cytometric analysis of propidium iodide (PI) stained cells. The results showed that compounds 1 and 2 induced a significant increase in the population of cells in G_0_/G_1_ phase and reductions in the S-phase population of cells treated with the corresponding IC_50_ concentration. Moreover, a significant increase was also observed in the population of cells in G_2_/M phase of HT-29 cells treated with compounds 1 and 2. The cell cycle results indicate that the compounds caused substantial arrest of cells in G_0_/G_1_ phase of the cell cycle. Cell cycle arrest at G_0_/G_1_ stage relates to the induction of cell cycle inhibitory proteins such as p16, p21, p27, which correlates with reduced expression of cyclins required for the G_1_ to S transition [31]. Liu et al. (2003) showed that terfenadine treatment induced upregulation of p53, p21/Cip1 and p27/Kip1 while simultaneous down regulation was observed for CDK2 and CDK4. Many anticancer drugs of natural origins such as paclitaxel, docetaxel, vinblastine and vincristine are known cell cycle inhibitors of the G_2_/M phase. Vinblastine and vincristine affect the G2/M phase of the cell cycle through inhibition of microtubule assembly while paclitaxel and docetaxel exert their actions through stabilization of microtubules, which induces the inhibition of microtubule disassembly and eventual arrest at G_2_/M phase of the cell cycle [32–34]. G_0_/G_1_ and G_2_/M arrest might be important pathways by which the isolated compounds induce cytotoxicity in cancer cells.

Agents that cause cell cycle arrest can be categorized into two classes: (1) those that directly inhibit DNA synthesis and (2) those that cause DNA damage leading to G_1_ or G_2_ arrest [35]. The effects of compounds **1** and **2** on DNA synthesis were evaluated using the cell proliferation ELISA chemiluminescent BrdU kit. The results showed that the two compounds significantly reduced cellular DNA synthesis within the duration of exposure to the IC_50_ levels in the respective cells. This result implies that the compounds can be placed in the category of agents that directly inhibit cellular DNA synthesis, which is consistent with the observed results in the cell cycle analysis with reduction of S-phase.

## Conclusion

This study described the isolation of two steroidal alkaloids from the *H. floribunda.* Even though these compounds demonstrated relatively high IC_50_ values in the cancer cells tested in this study, they can still be considered good candidates for the development of anticancer drugs since they were shown to have selective anti-proliferative activities against cancer cells. Moreover, These compounds can also be used as drug leads for the development of other drugs or they can be used in combination with other antineoplastic drugs to compliment the therapeutic effects. However, more in vitro and in vivo mechanistic studies are required to understand the full potential of these compounds.

## Abbreviations

ANOVA: Analysis of variance
BrdU: Bromodeoxyuridine
DMEM: Dulbecco’s Modified Eagle Medium
ELISA: Enzyme-linked immunosorbent assay
FBS: Foetal bovine serum
IC_50_: Half maximal inhibitory concentration
MTT: 3-[4, 5-dimethylthiazol-2-yl]-2, 5-diphenyltetrazolium bromide
PBS: Phosphate buffered saline
TMS: Tetramethylsilane
